# AI-Based Delirium Risk Prediction Compared With the Validated 4AT-Score Screening Tool in Patients With Trauma: Prospective Cohort Study

**DOI:** 10.2196/104907

**Published:** 2026-10-21

**Authors:** Klaus Franceschini, Marisa Valentini, Andreas Leithner, Diether Kramer, Stefanie Jauk, Lena Delia Lorenzer, Karin Hasiba-Seebacher, Stefan Sumerauer, Viktoria Christin Godschachner, Magdalena Hellauer, Gerald Sendlhofer

**Affiliations:** 1 Department of Orthopaedics and Trauma, Medical University of Graz, Graz, Styria, Austria, 43 31638581719; 2 Directorate for Digitalisation, IT, Organisational Development and Innovations, Styrian Hospital Holding, Graz, Austria; 3 Department of Psychiatry, Psychosomatics and Psychotherapeutic Medicine, Medical University of Graz, Graz, Austria; 4 Department of Neurology, Medical University of Graz, Graz, Austria; 5 Hospital Pharmacy, University Hospital of Graz, Styrian Hospital Holding, Graz, Austria; 6 Research Unit for Safety and Sustainability in Health Care, c/o Division of Plastic, Aesthetic and Reconstructive Surgery, Department of Surgery, Medical University of Graz, Graz, Austria

**Keywords:** delirium, AI, machine learning, 4AT, delirium screening, delirium prevention, clinical decision support, early delirium prediction

## Abstract

**Background:**

Delirium is a complex neuropsychiatric syndrome whose severe and lasting consequences in hospitalized patients are largely considered preventable. Prevention primarily relies on the early identification of high-risk patients prior to syndrome onset. However, this remains challenging in clinical practice due to the absence of a diagnostic gold standard and the considerable administrative burden associated with established screening tools such as the 4AT score.

**Objective:**

The aim of this study is to compare the 4AT score with an established AI-supported prognostic tool in applied clinical practice. Our goal is to demonstrate that shifting from a purely diagnostic approach toward AI-supported delirium risk prediction enables more efficient clinical workflows, reduces misclassification rates, facilitates earlier and more targeted interventions, and ultimately improves long-term patient outcomes.

**Methods:**

In this prospective study, a total of 2002 patients from a trauma ward were screened and assessed for delirium risk using the 4AT score and the AI-based tool at admission, on the evening of admission, and on the following day, with each assessment predicting the risk of delirium occurrence at any point during the remainder of the hospital stay. The AI tool based its predictions on clinical data automatically derived from electronic health records. The AI-based tool applied a previously developed and validated random forest model (not developed as part of this study).

**Results:**

Of the 2002 processed patients, the AI prediction tool correctly predicted 87.3% (165/189) of patients with delirium onset as at-risk, compared to an early detection rate of 28.0% (5/189) achieved by the 4AT score. Moreover, the AI-based tool correctly classified 73.9% (1340/1813) patients without delirium as not-at-risk, compared to 96.3% (1746/1813) for the 4AT score. Therefore, the AI-based tool achieved very good delirium predictions, with an area under the receiver operating characteristic curve of 0.884 (95% CI 0.8634‐0.9039).

**Conclusions:**

The AI-based delirium prognostic tool demonstrated superior early predictive capacity for delirium risk at admission, compared with the 4AT score. The AI-based tool enables proactive risk management and targeted risk reduction.

## Introduction

### Background

Delirium is an acute, temporary, and fluctuating disturbance of attention and cognition triggered by an underlying medical, neurological, or pharmacological condition [1]. With a frequency ranging from 29% to 64% in general medical wards, up to 50% following high-risk surgical procedures, and up to 75% in patients receiving mechanical ventilation in the intensive care unit, delirium is highly prevalent among hospitalized older adults [1-4].

Delirium onset follows dynamic vulnerability-stress models that integrate complex predisposing and precipitating factors. Baseline vulnerability includes preexisting cognitive impairments, affective comorbidities (advanced age), functional impairments (physical, vision, and hearing), and underlying biological frailty (such as sarcopenia) [1,5,6]. These baseline vulnerabilities interact with acute physiological stressors and organic causes: major surgery, polytrauma, infection/sepsis, dehydration, drops in hemoglobin level, dynamic fluctuations in anticholinergic burden resulting from inpatient pharmacotherapy [1,7], as well as prolonged fasting periods (potentially lasting several hours or even days, eg, due to delayed anesthesiology clearance in older patients or limited operating room capacity). Disorientation related to hospitalization itself plays an important role, especially if essential personal aids, such as hearing devices or glasses, are forgotten at the patient’s home or in their usual care environment at the time of acute admission [1].

Delirium itself is associated with an increased risk of mortality and long-term adverse outcomes, including a higher likelihood of developing dementia and requiring institutional care [8].

It is one of the major complications after surgery in older adults who are hospitalized. Furthermore, pharmacological treatments—such as antipsychotic medications—have often proven to be insufficient and ineffective in the management of delirium [9]. In contrast, multicomponent nonpharmacologic delirium prevention interventions have proven effective, reducing the incidence of delirium itself [10]. Furthermore, this multimodal nonpharmacologic approach has been associated with a trend toward a 0.33-day shorter length of stay and a 6% lower likelihood of institutionalization [10]. This is particularly relevant, given that 30% to 40% of delirium cases can be addressed before the syndrome’s onset [3,11].

### Standards and Prior Work

There is no gold standard tool available for delirium prediction with sufficient diagnostic accuracy [12]. The *DSM-5* (*Diagnostic and Statistical Manual of Mental Disorders* [Fifth Edition]) is commonly used as a reference standard [13]. *DSM-5* is seldom used in routine hospital practice due to its complexity, time, and the necessity of trained clinicians or certified psychologists [14]. Consequently, several delirium assessment tools with higher practical applicability and smaller range of items and points to assess were developed. These tools include the confusion assessment method [15], the Nursing Delirium Screening Scale [16], and the 4AT score [17].

Because preventing the onset of delirium is the primary goal, detecting at-risk patients is of utmost importance [3,11]. When direct prevention as exemplified by the Elder Life Program is not possible, early prediction is crucial [11]. The Elder Life Program includes an orientation protocol, vision protocol, hearing protocol, therapeutic activities, early mobilization, volume repletion, and a nonpharmacologic sleep protocol [11,18]. However, the effective implementation of delirium prediction, treatment, and prevention strategies is still a major challenge in health care all around the globe [9]. In general, strategies that solely rely on delirium prediction and subsequent management may be less effective than risk prediction approaches that allow for the identification of patients at high risk of developing delirium [19,20]. Numerous studies have shown that machine learning models generally outperform rule-based approaches; however, clinical validation within the context of delirium risk prediction is still needed [20-22].

A scoping review identified the following key challenges [23]. First, the underlying dataset frequently lacks unstructured clinical data, which would theoretically require context-sensitive transformer models with strong protections against label leakage for adequate processing [24]. Furthermore, many models rely exclusively on static electronic health record (EHR) snapshots, without accounting for the temporal dynamics of patient physiology [24]. Another recurring issue across included studies is the predominant use of critically ill patient cohorts, limiting broader clinical applicability across more heterogeneous populations. Furthermore, while many of the tools reviewed demonstrated promising predictive performance, the majority have not been prospectively validated or adapted under real-world clinical conditions. Notably, predictive performance frequently degrades when models are applied to patient populations that differ from the original training cohort with respect to age, sex, and comorbidity distribution, underscoring the critical importance of site-specific calibration prior to clinical deployment [24].

Common challenges associated with AI-based tools in medicine frequently concern the collection of data required for model development, as well as the limited availability or restricted access to such data. Furthermore, concerns regarding data security and patient privacy are frequently raised. The volume of data, as well as specific inclusion and exclusion criteria applied to the datasets used for training AI models, may introduce bias. One of the most prominent criticisms, addressed in a 2021 review, is the “black box” problem inherent to many AI systems: such tools are often unable to provide transparent reasoning for the predictions they generate [25].

The aim of this paper is to compare a well-established delirium screening approach (4AT score) with an AI-supported prognostic tool designed to predict patients at high risk at an early stage. Second, the goal of this study is to demonstrate that shifting from a purely questionnaire-based screening approach toward AI-supported delirium risk prediction could enable efficient achievements, including optimized clinical workflows, reduced misclassification, and earlier and more targeted interventions, possibly leading to improved long-term patient outcomes.

The current study is part of a multiprofessional project established to validate the AI-based delirium prediction tool and to implement multicomponent interventions for the prevention of delirium in an Austrian university hospital.

## Methods

### Recruitment

All acute and elective patients admitted to a university hospital trauma ward were included in the study between June 1, 2024, and May 31, 2025.

No formal sample size was calculated, and all eligible patients admitted during the prespecified 12-month period were included to maximize the number of observed delirium events.

For improved readability, delirium onset/diagnosis during the inpatient stay will hereafter be referred to as acute delirium.

### Delirium Labeling

For delirium labeling, the EHR was searched for delirium-related codes, specifically F05 (delirium, not induced by alcohol or other psychoactive substances) and F10.4 (mental and behavioral disorders due to use of alcohol: withdrawal state with delirium). In addition to *ICD (International Classification of Diseases*) code–based identification, the labeling process also incorporated physician discharge letters documenting a clinical delirium diagnosis in narrative form (ie, noncoded diagnoses without a corresponding *ICD* code), nursing diagnoses of acute confusion (similarly without a formal *ICD*-coded diagnosis), psychiatric consultation notes, and cases in which patients had received a coded F05 diagnosis during a prior hospitalization. Regarding the latter criterion, the decision was made on the basis that, as the primary focus of this study is risk prediction, patients with a history of prior delirium are at substantially elevated risk of experiencing a subsequent episode and are therefore classified as high-risk patients by default [26]. For the 21 patients classified as positive for delirium based solely on a documented diagnosis of delirium during a previous hospital admission, this approach may introduce a degree of circularity, as delirium history was also incorporated as a predictor in the AI model. Therefore, a sensitivity analysis excluding these 21 cases was additionally performed to test the robustness of the model’s performance.

The method of outcome ascertainment (*ICD* coding, discharge letters, psychiatric consultations, and nursing diagnoses) was applied uniformly across the entire patient population, irrespective of age, sex, or other sociodemographic characteristics.

### Screening Tool—4AT Score

The 4AT is a specific screening tool for delirium and is routinely used [17]. The resulting score consists of 4 components. Component 1 evaluates the patients’ level of alertness. Components 2 and 3 are brief cognitive screening tests: the Abbreviated Mental Test 4 and the Months Backwards test (attention assessment). Component 4 assesses acute changes or fluctuations in mental status (Table 1). The total 4AT score ranges from 0 to 12. A score of 0 indicates that delirium and/or moderate-to-severe cognitive impairment is unlikely. Scores of 1 to 3 may reflect significant cognitive impairment. A score of 4 or higher indicates possible delirium [17]. During the study, assessments were carried out on multiple occasions: at the time of admission, on the evening of the day of admission, and on the second day of admission. Furthermore, repeated 4AT assessments were conducted in patients with elevated scores (≥4) or in cases where clinical deterioration was observed. However, for the purpose of this study and to ensure a comparable statistical approach with the prediction tool, only the highest score obtained on the day of admission was used for analysis. All 4AT assessments were performed by ward nursing staff.

**Table 1.** Reproduced from the 4AT website, licensed under CC BY 4.0 [27].

Item and criteria	Score
Alertness	
Normal (fully alert, but not agitated, throughout assessment)	0
Mild sleepiness for <10 seconds after waking, then normal	0
Clearly abnormal	4
Abbreviated Mental Test-4: age, date of birth, place (name of the hospital or building), current year	
No mistakes	0
1 mistake	1
2 or more mistakes/untestable	2
Attention: months of the year backward	
Achieves 7 months or more correctly	0
Starts but scores<7 months/refuses to start	1
Untestable (cannot start because unwell, drowsy, and inattentive)	2
Acute change or fluctuating course	
No	0
Yes	4

### AI-Supported Prognostic Tool

#### Clinical Implementation and Risk Stratification

The AI-based Personalized Risk Tool was developed and provided by Predicting Health GmbH [20]. It predicts individual delirium risk of patients, enabling more efficient allocation of health care resources. Clinical data were derived from EHRs by integrating defined clinical, laboratory, pharmacological, and transfer data. The dataset comprised both demographic and clinical information extracted from EHRs. Demographic and admission data included patient age, sex, as well as the ward and date/time of hospital admission. Medical data included *ICD-10* (*International Statistical Classification of Diseases, Tenth Revision*)–coded diagnoses, and laboratory data comprised standardized laboratory values (LOINC [Logical Observation Identifiers Names and Codes] codes) [28]. Medication data such as prescribed drugs were encoded using the Anatomical Therapeutic Chemical classification system [28]. Additional clinical features included nursing assessments, such as documented visual or hearing impairment, hypoactive behavior, and the use of sedative medications [28]. Procedures/surgeries were coded according to the Austrian procedure classification system. Hospital flow variables, including counts and times of ward transfers, were also recorded [28].

The underlying machine learning models were trained on a cohort of more than 19,000 inpatients using the random forest architecture. The outcome variable was defined using the *ICD-10* codes F05 and F10.4 to identify patients with delirium. The models were trained using a 75%/25% train-test split implemented via the caret package [29] (version 6.0-86; Max Kuhn), with an up-sampling method and 10-fold cross-validation. Predictions were exclusively generated using data available up to the designated prediction time points, thereby preventing future hospital data from leaking into the risk prediction. The most important features in the random forest models included age, number of previous hospital stays, Charlson Comorbidity Index, and nurse-assessed orientation. Subgroup bias analyses demonstrated comparable prediction accuracy between male and female cohorts, indicating no performance disparities. Further details on feature importance and model training are described in our previous research [20].

The algorithm processes only routinely documented EHR data. Predictions are automatically generated at admission and recalculated on subsequent evenings to incorporate newly available clinical data. Patients with a delirium code F05 from a previous hospital stay are assigned the highest risk score and do not undergo risk prediction, as a history of delirium is highly associated with the current risk of delirium [20].

As the primary development of the AI-based tool is not the focus of this paper, further details, such as predictor preselection, hyperparameter tuning, model architecture rationale, prediction calculation methodology, and the rationale for class-imbalance handling during training, are omitted here. These aspects are described in greater depth in the developers’ publications [28]. This study primarily focuses on the analysis of the independent cohort.

When evaluated prospectively in real-world clinical workflows, the AI-supported tool demonstrated robust and stable discriminative performance. This was demonstrated already in the initial broad multidepartment evaluation with an area under the receiver operating characteristic curve (AUROC) of 0.855, as well as in a specialized surgical validation cohort with an AUROC of 0.883 [20,28].

The delirium risk prediction tool was used to run locally in the background on the hospital information system (HIS), generating automated risk estimates using random forest models. Based on the prevalence of delirium and the currently available resources for prevention, the top 5% of patients with the highest predicted risk were categorized as “very high risk,” the subsequent 10% as “high risk,” and the remaining 85% as “low risk.” In other words, the model cutoffs for the risk classes were defined at the 85th and 95th percentiles of the predicted risk distribution [20]. These predictions were based on EHR-derived features and calculated at 3 distinct time points: upon admission, on the evening of admission, and on the evening of the second hospital day. For the analysis and comparison, only the value from the first evening of admission was used. Risk levels were stratified into 3 groups, that is, low, high, and very high [28].

#### User Surface

Two methods were used to present the calculated risks. In the clinical workspace of the HIS, a red or yellow icon indicated “very high risk” (95th-100th percentile) or “high-risk” (85th-94th percentile) patients, respectively, with no symbol displayed for “low-risk” patients [20]. Patient-specific information could be accessed via an R Shiny web application (Posit Software, PBC) by clicking the icon or empty cell, displaying demographic, clinical, and additional data for each patient (Figures 1 and 2). Detailed information about the use and surface is provided in prior work [20].

**Figure 1.**
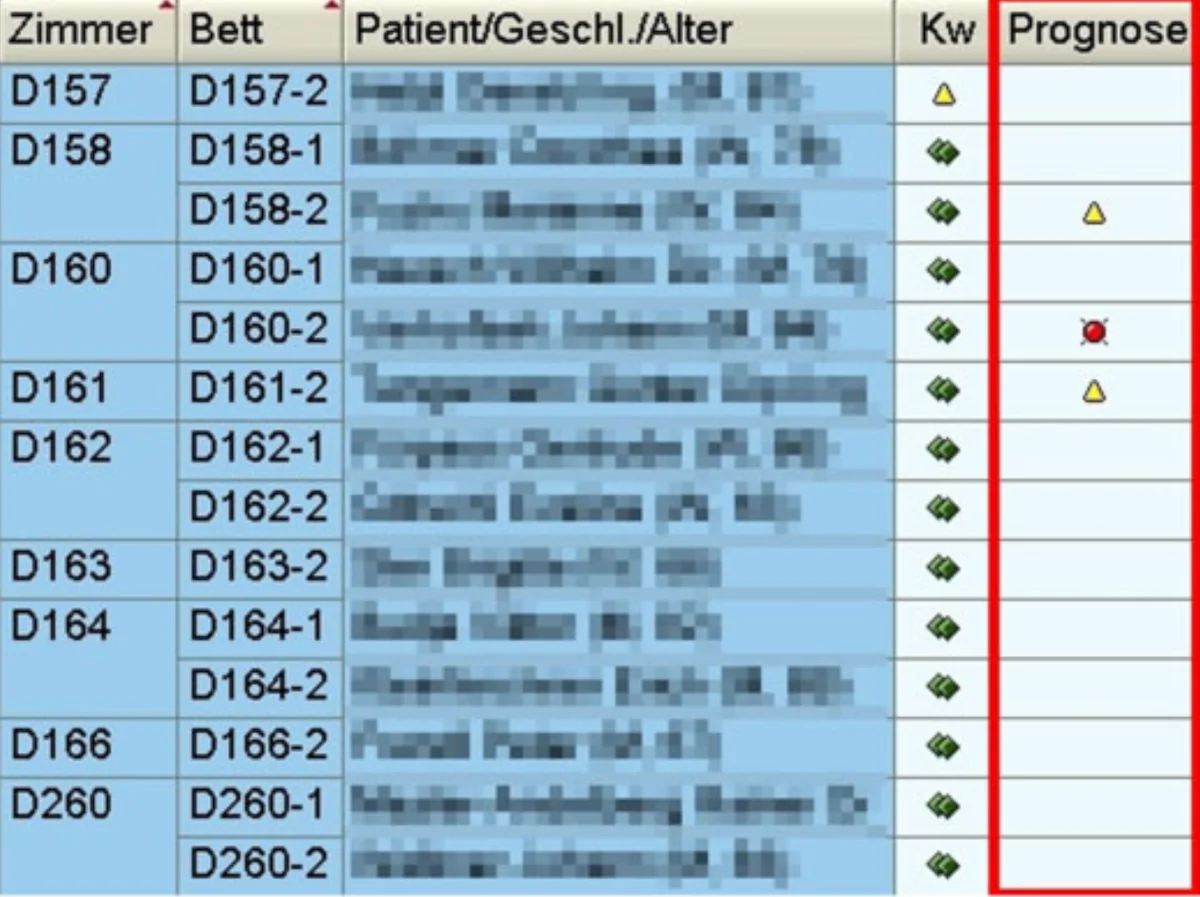
Delirium risk presented as color-coded icons (red: “very high risk,” yellow: “high risk”) in the electronic ward occupancy overview [20].

**Figure 2.**
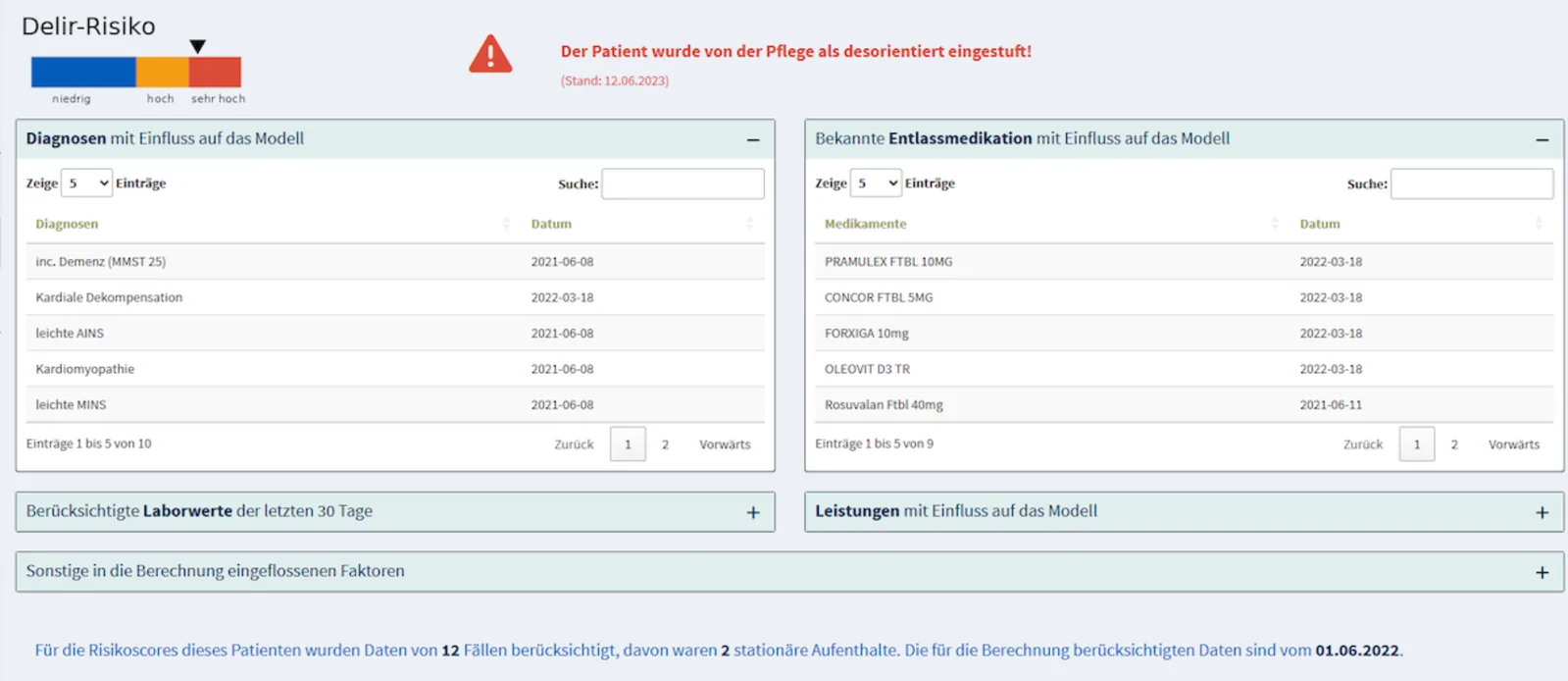
Web application visualizing features used in the random forest model. (Top) AI-based delirium risk (“very high,” ie, red); (bottom) relevant diagnoses, procedures performed, laboratory results, and nursing assessment data in the 4 boxes [20].

**Figure 3.**
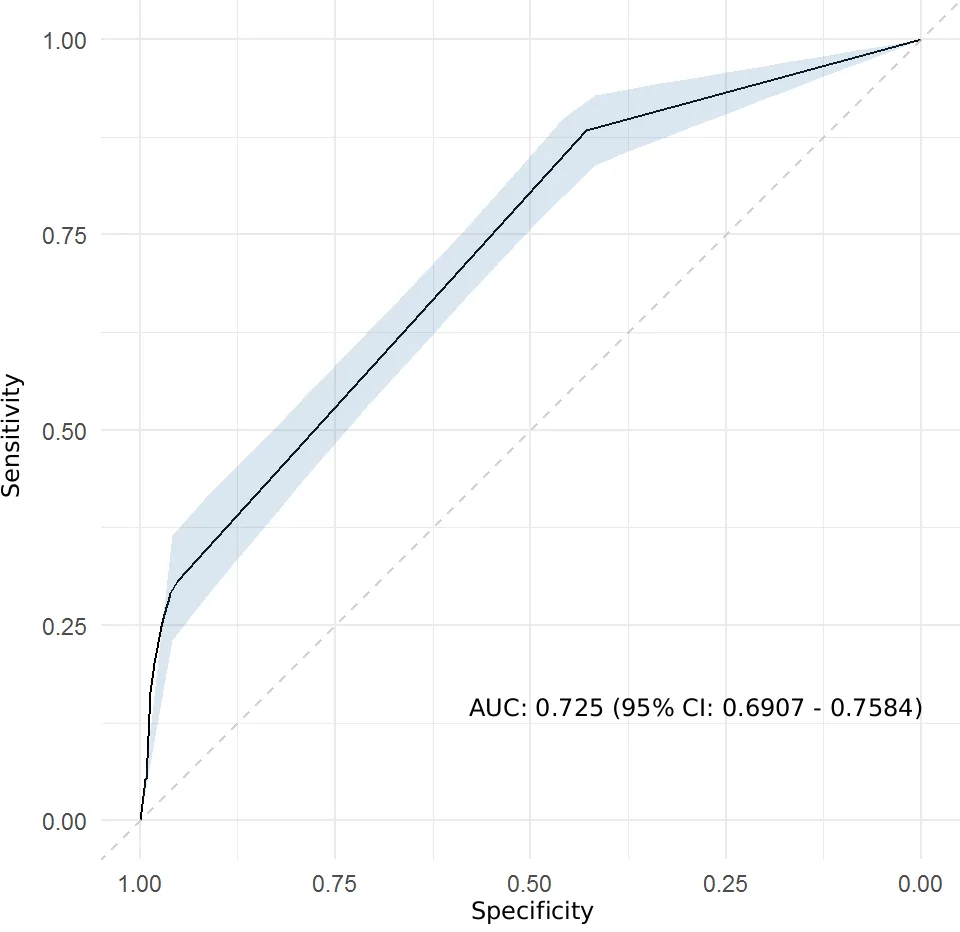
Area under the receiver operator characteristic curve (AUROC) of the highest 4AT score of the day of admission vs acute delirium.

### Statistical Analysis

#### AI-Based Tool

As the algorithm of the Personalized Risk Tool grouped patients into 3 risk categories, the “high risk” and “very high risk” groups were combined and treated as at-risk for delirium (ie, delirium positive). Based on this binary classification, sensitivity, specificity, and positive and negative predictive values were calculated for the AI prediction algorithm [28].

#### 4AT Score

For the 4AT score, the results were grouped into 3 categories: 0, 1‐3, and ≥4 points. Only the category with scores ≥4 was classified as delirium positive, while the other 2 were combined and considered delirium negative. Again, based on this binary classification, sensitivity, specificity, and positive and negative predictive values were calculated for the 4AT Score.

The AI model and 4AT score performances were evaluated using receiver operating characteristic curves with 95% CIs computed via the DeLong method, calibration plots, and calculations of sensitivity, specificity, and predictive values [30]. All analyses were performed using R version 3.6.2 (“Dark and Stormy Night;” R Foundation for Statistical Computing).

### Data Preparation

EHR data required for the analysis were extracted from the HIS. This included information used for delirium labeling (corresponding *ICD-10* codes, discharge letters, psychiatric consultations, and nursing diagnoses), demographic data such as age and sex, as well as transfer data for all patients included in the analysis. Individual risk predictions generated by the Personalized Risk Tool were extracted from the on-premises database and linked to the study cohort. All 4AT scores were available from the HIS. Patients with a missing 4AT score or a missing AI-based risk prediction were excluded from the analysis.

### Ethics Considerations

The study was approved by the local institutional ethical review board (reference number 30‐146 EX 17/18).

## Results

Of the 2003 patients assessed, one patient was excluded due to a missing 4AT score on the day of admission. No further missing data were identified in the analyzed dataset. A total of 168 patients with delirium diagnosed during the inpatient stays and 21 patients with status post delirium with delirium diagnoses in prior cases were assessed (n=189 patients with delirium). Demographic data show a clear age-dependent increase in delirium incidence. With respect to sex, the incidence of delirium is higher in men (97/1013, 9.6%) than in women (92/989, 9.3%; Table 2).

**Table 2.** Distribution of delirium in patients based on age and sex.

Distribution	Sex, n/N (%)		Age group (y), n/N (%)				Total (n)
	Male	Female	<50	50‐64	65‐80	>80	
No delirium	916/1013 (90.4)	897/989 (90.7)	561/568 (98.8)	393/415 (94.7)	427/469 (91.0)	432/550 (78.6)	1813
Delirium	97/1013 (9.6)	92/989 (9.3)	7/568 (1.2)	22/415 (5.3)	42/469 (9.0)	118/550 (21.5)	189
Total	1013/2002 (50.6)	989/2002 (49.4)	568/2002 (28.4)	415/2002 (20.7)	469/2002 (23.4)	550/2002 (27.5)	2002

Of these 189 patients with delirium, 8 carried a formally coded *ICD* diagnosis of F05 (delirium, not induced by alcohol or other psychoactive substances). No cases were coded as F10.4 (alcohol withdrawal syndrome with delirium). The remaining 181 cases were documented without an *ICD* code: 62 cases were documented in the physician’s discharge letter (noncoded diagnosis, without a corresponding ICD code), 44 cases were recorded as nursing diagnoses of acute confusion (again without a formal *ICD*-coded diagnosis), 54 cases were identified through psychiatric consultations in which delirium was diagnosed, and 21 cases involved patients with a coded F05 diagnosis of delirium (delirium, not induced by alcohol or other psychoactive substances) during a previous hospital stay.

The 4AT score was available for all 2002 (100%) patients, in which 120 patients had a score of ≥4 points and 1882 patients had a score of 0 to 3 points. For the selected patients with a score of ≥4 (Table 3), we also recorded on which day of the week the score was recorded (corresponding to the evening of the patient’s admission day). Regarding the distribution across weekdays, Thursday, Monday, and Wednesday recorded the highest counts with 62, 61, and 59 patients, respectively. Tuesday, Friday, and Sunday showed an intermediate range of approximately 51 (±1) patients, whereas Saturday exhibited the lowest count with 41 patients.

**Table 3.** Comparison of highest 4AT-score values with acute delirium during the hospital stay.

Distribution of delirium	4AT screening score, n/N (%)			Total, n/N (%)
	0	1-3	≥4	
No delirium	776/1813 (42.8)	970/1813 (53.5)	67/1813 (3.7)	1813/2002 (90.6)
Delirium	22/189 (11.6)	114/189 (60.3)	53/189 (28.0)	189/2002 (9.4)
Total	798/2002 (39.9)	1084/2002 (54.2)	120/2002 (6.0)	2002/2002 (100)

Among the 1813 patients without delirium, the 4AT score correctly screened 96.3% (1746/1813) of patients. On the other hand, among the 189 patients with delirium, the 4AT score correctly (score ≥4) detected only 28.0% (53/189) of patients (Table 3). The AI prediction tool output was available for all patients (N=2002, 100%).

**Table 4.** Comparison of the AI-scored risk at the day of admission with the occurrence of acute delirium during the hospital stay, as well as with acute delirium or a 4AT score ≥4 during the stay.

Distribution of delirium	AI-based tool delirium prediction, n/N (%)			Total, n/N (%)
	Low risk	High risk	Very high risk	
No delirium	1340/1813 (73.9)	283/1813 (15.6)	190/1813 (10.5)	1813/2002 (90.6)
Delirium	24/189 (12.7)	59/189 (31.2)	106/189 (56.1)	189/2002 (9.4)
Total	1364/2002 (68.1)	342/2002 (17.1)	296/2002 (14.8)	2002/2002 (100)
No delirium and 4AT score <4	1287/1605 (80.2)	208/1605 (13.0)	110/1605 (6.9)	1605/2002 (80.2)
Delirium or 4AT score ≥4	77/397 (19.4)	134/397 (33.8)	186/397 (46.9)	397/2002 (19.8)
Total	1364/2002 (68.1)	342/2002 (17.1)	296/2002 (14.8)	2002/2002 (100)

A high or very high delirium risk was predicted in 31.8% (638/2002) of patients and a low delirium risk in 68.1% (1364/2002) of patients.

Among the 1813 patients without delirium, the AI model correctly predicted 73.9% (n=1340) of patients as not-at-risk, while among the 189 patients with delirium, the AI tool correctly detected a high or very high risk in 87.3% (n=165) of patients (Table 4). When excluding the 21 patients with status post delirium, the AI model correctly identified 85.7% (144/168) of the patients with delirium.

The AUROC of the highest 4AT score on the day of admission calculated vs the delirium onset or diagnosis during the inpatient stay was 0.725 (95% CI 0.6907‐0.7584; Figure 3).

The AUROC of the AI prediction tool categorization on the day of admission, calculated vs the delirium diagnosis during the inpatient stay, was 0.884 (95% CI 0.8634‐0.9039; Figure 4).

**Figure 4.**
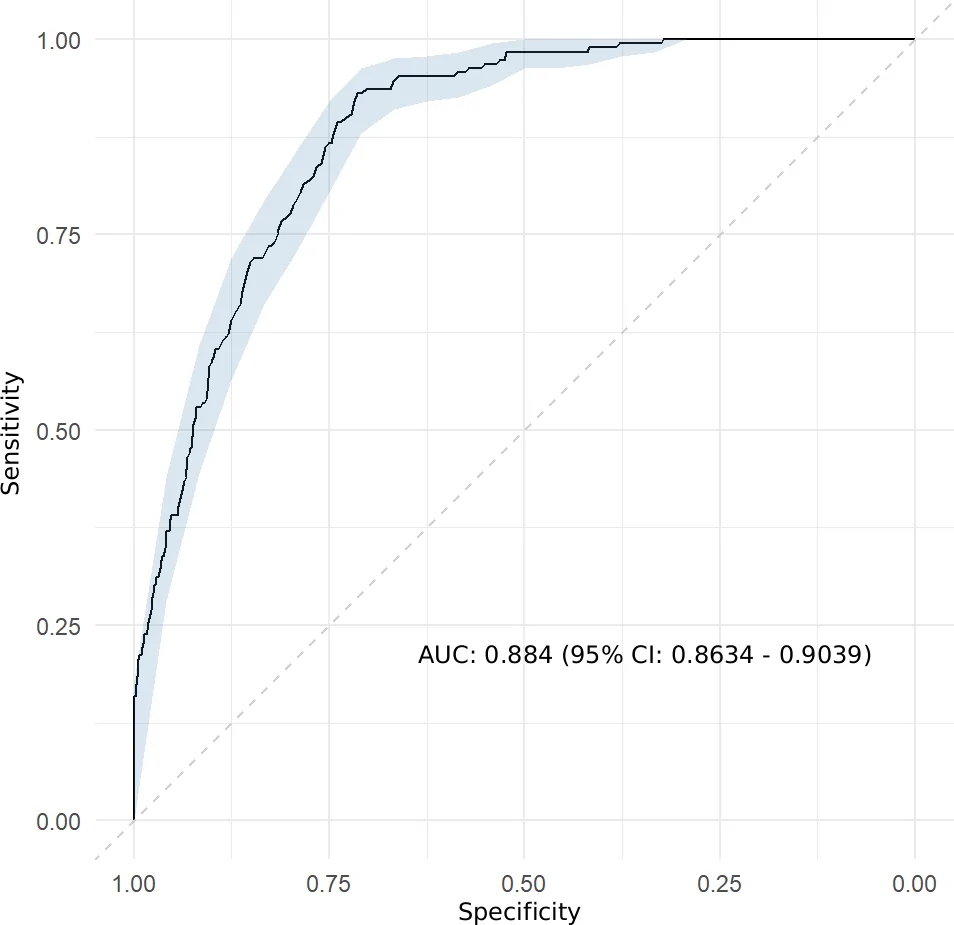
Area under the receiver operator characteristic curve (AUROC) of the AI-based tool categorization vs delirium diagnosis during the inpatient stay.

The 2 CIs (Figures 3 and 4) do not overlap. The difference in predictive ability between the models is statistically significant.

When extending the comparison of the AI-based tool to include patients with in-ward delirium or a 4AT score ≥4, the model demonstrated the following risk-predictive performance: among the 1605 patients without delirium, the model correctly classified 80.2% (1287/1605) of patients. Among the 397 patients with delirium, the model correctly assigned a high-risk or very high-risk category in 80.6% (320/397) of patients (Table 3).

The AUROC of the AI prediction tool categorization on the day of admission, calculated vs the delirium diagnosis during the inpatient stay or a 4AT score of ≥4, was 0.883 (95% CI 0.8652‐0.899; Figure 5).

**Figure 5.**
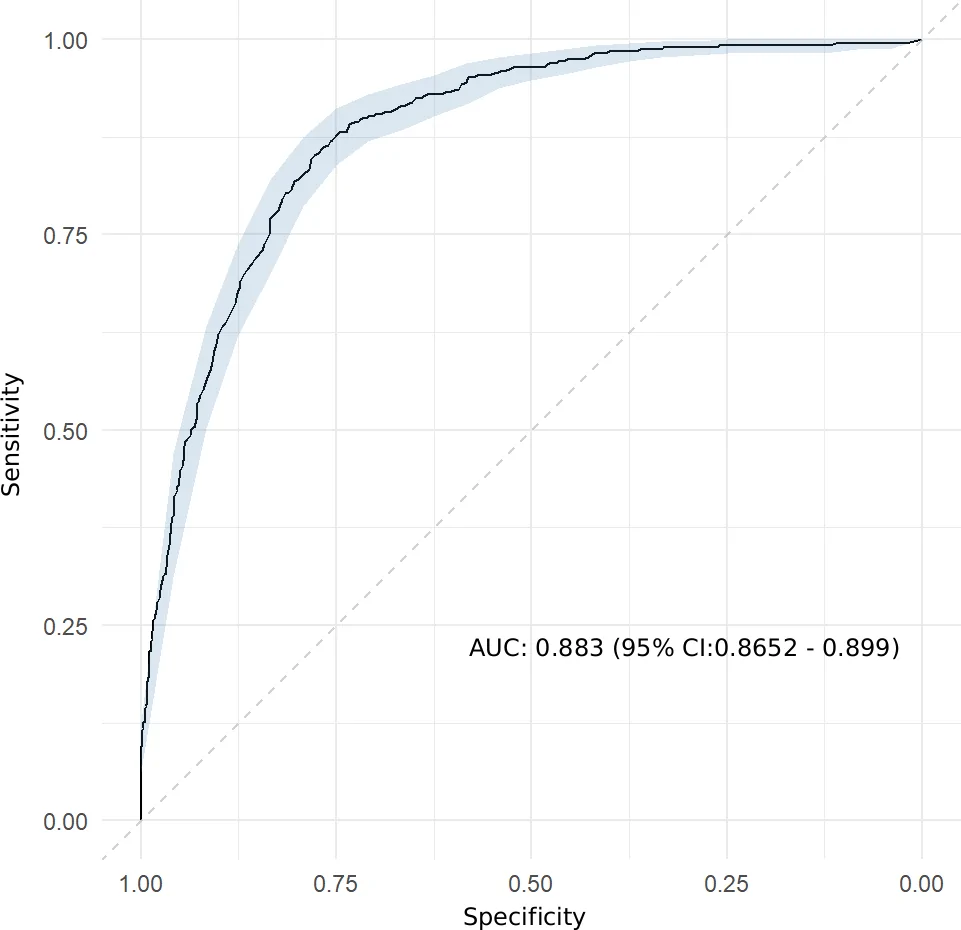
Area under the receiver operator characteristic curve (AUROC) of the AI-based tool vs delirium diagnosis during the inpatient stay or 4AT score ≥4.

## Discussion

### Interpretation of Findings

We aimed to test and evaluate an AI-based risk prediction tool in routine clinical practice and to compare its performance against a well-established diagnostic screening instrument (4AT score).

The risk prediction demonstrated a sensitivity of 87.3%, while the 4AT score showed a sensitivity of 28.0%. Regarding patients without delirium, the AI tool’s prediction showed a specificity of 73.9%, compared to the specificity of 96.3% achieved by the 4AT score. The discriminative performance of the AI-based tool achieved an AUROC of 0.884, indicating excellent discrimination, with an 88.4% probability of correctly categorizing patients developing delirium as at-risk. The narrow CI detected showed high statistical precision. Additionally, given that the entire interval substantially exceeds the 0.8 threshold, the result can be considered robust and clinically meaningful. In comparison, the 4AT score shows a notably lower AUROC, which reflects acceptable but moderate performance as an early detection tool. As the 2 CIs do not overlap, the first model demonstrates clear superiority.

### Limitations

This study has several limitations inherent to the analyzed topic and study design. The most important limitation is the fundamental difference in purpose between the 2 instruments evaluated. The 4AT score is a validated diagnostic screening tool intended to detect current delirium or acute cognitive fluctuations, whereas the AI-based model is a prognostic tool designed to estimate the risk of developing delirium during hospitalization. In the present study, we compared the 4AT score’s performance with an AI-based prognostic tool: a reference standard was required against which the performance of the AI-based prediction model could be benchmarked. In the absence of a more suitable comparator, the well-established 4AT score served this purpose. Moreover, the 4AT questionnaire is, by definition, a screening instrument designed to assess the patient’s current delirium status, but it also allows to observe fluctuations and changes in the patient’s neurological status over time and should be interpreted as such. In the context of this study, the 4AT score was evaluated under conditions that may not fully reflect its intended use in clinical practice, as only the highest score on the day of admission was considered.

Consequently, the 2 approaches address different clinical questions and should be regarded as complementary rather than directly competitive. This study’s results suggest that the AI-based approach may provide greater predictive value at the time of admission for identifying patients at risk of developing delirium during hospitalization, while the 4AT remains an important tool for ongoing clinical screening during the hospital stay.

Furthermore, the performance of the AI model is dependent on the quality, completeness, and consistency of the underlying clinical data. Variability in documentation and coding quality represents a key success or failure factor for the AI-based tool, as its predictions are inherently dependent on the accuracy and consistency of the underlying clinical data in the HIS. This could have led to documentation, selection, and cotreatment biases in the present study.

A total of 638 (31.8%) patients were classified as having a high or very high risk by the AI-based tool. Of these, 473 patients did not develop delirium during the hospital stay. This corresponds to a false-positive rate of 26.1%. However, this does not imply that these patients were not at risk of delirium. In some cases, precipitating factors may have been absent during the current hospital stay, and, consequently, the patients did not develop delirium. Moreover, the ultimate goal of such a prognostic AI-based tool is to trigger targeted multidisciplinary preventive measures and, ultimately, to minimize the onset of delirium. Therefore, if an increasing number of at-risk patients do not develop delirium and the false-positive rate increases, the aim of such an AI-based prognostic model is achieved. However, the impact of such risk stratification through possible targeted preventive interventions was not evaluated in the present study.

Although the absence of a high or very high delirium risk according to AI risk stratification does not imply the absence of delirium risk, alert fatigue is a crucial aspect to consider and may evolve over time. Resources in hospital wards are limited, and it may not be feasible to implement preventive measures for 31.8% (n=638) of patients in a ward. In such cases, the AI-based tool could be recalibrated, and the number of patients assigned to each risk category could be adapted. This could reduce the number of risk alerts.

The cohort analyzed differed from the development cohort with respect to center composition. The evaluation cohort was derived from a single center, whereas the development cohort was based on multiple centers. The development dataset relied on *ICD-10* coding, whereas the evaluation dataset additionally incorporated noncoded clinical documentation (discharge letters, nursing diagnoses, and psychiatric consultations) to identify cases of delirium.

### Comparison With Prior Work

A systematic review and meta-analysis from the year 2022 for the 4AT score show a pooled sensitivity and specificity of 87% and 87%, respectively. In combination with its conciseness and simplicity, it confirms itself as an established and useful tool [23].

As for the AI, a systematic review and meta-analysis of the year 2022 for machine learning–based prediction models for delirium show that the overall pooled AUROC for predicting delirium was 89%, with sensitivity of 85% and specificity of 80% [31].

Compared to the pooled values reported, the AI tool used in this study demonstrated a higher sensitivity, exceeding the pooled values reported in the recent meta-analysis. This, however, comes at the trade-off of lower specificity.

Notably, the 4AT score performed considerably below the values reported in prior studies [17,23], which is attributable to the fact that only the single highest value recorded on the day of admission was used in this analysis (as a benchmark for the AI model). Of note, the 4AT score is, by definition, a screening instrument designed to assess the patient’s current delirium status and is used multiple times a day to capture fluctuations. Therefore, these results do not call into question the quality of the 4AT score.

### Advantages and Disadvantages (Early Clinical Detection and AI Prediction Model)

It should be noted that the 4AT score is a questionnaire-based tool, which considerably increases the costs and infrastructure associated with its administration. From a practical standpoint, the AI-based tool offers easier implementation in the overall workflow than the 4AT score. While the 4AT score requires active administration by nursing staff, followed by manual documentation and transfer into the EHR, the AI-based tool operates automatically in the background, functioning both as a decision-support tool and a source of clinical information. On the other hand, the AI-based tool grounds its predictions and prognoses on the information documented (correctly) in the HIS. Therefore, a drawback for the AI-based tool is the absence of standardized diagnostic criteria for delirium, resulting in *ICD*-coding being applied less consistently in patients with confirmed delirium compared to other medical conditions. One contributing factor is that delirium diagnoses often rely on subjective clinical assessment of the patient’s condition, a challenge that is further compounded by the varying subtypes (hypoactive, hyperactive, and mixed forms) and severity levels of delirium [20,26].

In general, other studies addressing AI-based delirium diagnostics using EHR data continue to face certain limitations, several of which are at least partially addressed by our tool.

A notable strength of the present study (in contrast to the majority of comparable prior studies) is that the AI prediction tool was validated and tested under real-world clinical conditions.

Nevertheless, further studies that include varying cultural backgrounds and differing health care systems remain warranted. AI models have the potential to support health care providers in their daily clinical tasks. By assuming a variety of responsibilities, AI-based tools may contribute to a reduction in overall workload, enabling providers to concentrate their efforts on a more focused set of tasks [25].

### Conclusions

The AI-based delirium prognostic tool demonstrated superior early predictive capacity for delirium risk at admission, compared to the 4AT score. Importantly, these 2 approaches address distinct clinical questions and should therefore be considered complementary rather than directly competing. This distinction is particularly relevant because no sufficiently comparable, well-established prediction tool currently exists against which this AI-based tool can be directly evaluated. A notable strength of the present study was that the AI prediction tool was validated and tested under real-world clinical conditions. The AI-based tool showed high sensitivity, exceeding values reported in prior meta-analyses, but with a trade-off in specificity [31]. The resulting insights may enable proactive risk management and targeted risk reduction.

## Supplementary material

10.2196/104907 Checklist 1 TRIPOD-AI checklist.

## Abbreviations

AUROC: area under the receiver operating characteristic curve
*DSM-5*: *Diagnostic and Statistical Manual of Mental Disorders* (Fifth Edition)
EHR: electronic health record
HIS: hospital information system
*ICD*: *International Classification of Diseases*
*ICD-10*: *International Statistical Classification of Diseases, Tenth Revision*
LOINC: Logical Observation Identifiers Names and Codes
